# Anti‐Amyloid Prescribing in Alzheimer's Disease: Ohio State University Clinical Guidelines for Patient Selection, Assessments, and Management

**DOI:** 10.1002/alz70858_101678

**Published:** 2025-12-25

**Authors:** Douglas W. Scharre, Jessica Truelove, Renee Kovesci, Arun Ramamurthy, Kristina Thurin, Ashley Middleton, Soumya Bouchachi

**Affiliations:** ^1^ Ohio State University Wexner Medical Center, Columbus, OH, USA

## Abstract

Anti‐amyloid monoclonal antibodies are approved for use in patients with Mild Cognitive Impairment due to Alzheimer's disease (MCI‐AD) or mild AD dementia (ADD). In real world settings, the importance of efficient early‐stage identification and careful patient selection, is critical for optimal outcomes and reduction of risks when using anti‐amyloid therapies (AAT). We developed, at The Ohio State University, clinical guidelines for prescribing AAT including the identification of appropriate patients, required assessments, treatment, and monitoring. Our guideline selection criteria for appropriate patients require a change in memory or thinking over at least 6 months and a clinical diagnosis of MCI‐AD or mild ADD which was operationally defined by specific cognitive assessment score cut offs (SAGE or BrainTest >9, MMSE>19, or MoCA >16), number of cognitive domains impaired (≥1 or ≤4 domains of memory, executive, attention, language, and visuospatial), and functional surveys that require hands‐on assistance for one to four instrumental activities of daily living (ADLs) and the ability to perform all basic ADLs. Our guidelines required ruling out other non‐Alzheimer's causes of cognitive impairment and verifying amyloid pathology by FDA approved AD biomarkers. Patients were excluded if they were on anticoagulants, unable to have a brain MRI, or with significant psychiatric symptoms making infusions unsafe or impractical. Our guidelines specify every 6‐month cognitive and functional assessment monitoring as above with stoppage criteria for ATT including upon progression to moderate ADD stage. After 18 months of therapy, and every 6 months thereafter if they remain on donanemab therapy, our guidelines recommend that amyloid pathology should be reevaluated and if levels are normal, donanemab should be stooped. Lecanemab can be continued until progression to moderate ADD stage. Our guideline includes the FDA prescribing information regarding recommended infusion dosing and timing for lecanemab and donanemab, titration schedule for donanemab, and MRI safety monitoring. We present these clinical guidelines for ATT prescribing as an efficient way to identify appropriate patients and monitor them in real world practice settings. We encourage debate and alternative guidelines that are data‐driven following clinical and real‐world investigations, with the goal of improving on this guideline we presented.

